# Immunotherapy for Gastroesophageal Cancer

**DOI:** 10.3390/jcm5100084

**Published:** 2016-09-22

**Authors:** Emily F. Goode, Elizabeth C. Smyth

**Affiliations:** The Royal Marsden Hospital, NHS Foundation Trust, London SW3 6JJ, UK; Emily.goode@rmh.nhs.uk

**Keywords:** anti-PD-1, anti-PD-L1, checkpoint inhibitor, ipilimumab, esophageal cancer, gastric cancer, immunotherapy, pembrolizumab, nivolumab

## Abstract

Survival for patients with advanced oesophageal and stomach cancer is poor; together these cancers are responsible for more than a million deaths per year globally. As chemotherapy and targeted therapies such as trastuzumab and ramucirumab result in modest improvements in survival but not long-term cure for such patients, development of alternative treatment approaches is warranted. Novel immunotherapy drugs such as checkpoint inhibitors have been paradigm changing in melanoma, non-small cell lung cancer and urothelial cancers. In this review, we assess the early evidence for efficacy of immunotherapy in patients with gastroesophageal cancer in addition to considering biomarkers associated with response to these treatments. Early results of Anti- Programmed Cell Death Protein-1 (anti-PD-1), anti-PD-L1 and anti-Cytotoxic T-lymphocyte assosciated protein-4 (anti-CTLA4) trials are examined, and we conclude with a discussion on the future direction for immunotherapy for gastroesophageal cancer patients.

## 1. Introduction

Recent advances in immunotherapy have been transformational in the treatment of metastatic melanoma; as a result, immunotherapy is being explored in many other cancers [1,2]. Emerging data suggest that anti-PD-1 therapy may be effective in a subset of gastroesophageal cancers [3]. These encouraging results prompt this review in an attempt to outline the interface between current treatment paradigms, the biology of gastroesophageal cancer, and early trial results in order to better understand where immunotherapy might be best poised to help patients diagnosed with this disease.

## 2. Gastroesophageal (OG) Cancer: Epidemiology and Current Treatment Patterns

Gastric and oesophageal cancers are leading causes of cancer mortality globally; they are the 5th and 8th most common cancers diagnosed worldwide, and are responsible for a combined total of 1,407,000 new cases and 1,123,000 deaths annually [4]. Whilst the incidence of distal gastric adenocarcinoma has been falling for many years, the number of patients diagnosed with proximal gastric cardia and gastroesophageal junction cancers has increased in many developed countries [5]. These opposing findings are respectively attributed to improvements in diet and control of chronic *H. pylori* infection, and an increase in risk factors such as gastroesophageal reflux disease and obesity [6].

The majority of patients with gastric or oesophageal cancer are diagnosed at a locally advanced or advanced stage when surgical treatment is not an option. Systemic chemotherapy remains the primary mode of treatment for advanced disease and has been shown to improve survival when compared to supportive care; however, even with optimal chemotherapy median survival for fit patients treated on first line clinical trials is 9–11 months [7,8]. Worldwide, a combination of a platinum and fluoropyrimidine chemotherapy doublet with or without the addition of an anthracycline or taxane is considered a standard first-line treatment for patients with human epidermal growth factor receptor-2 (HER2) negative advanced OG cancer. For patients with HER2 positive gastroesophageal cancer (~20%), the landmark 2010 Trastuzumab for Gastric Cancer (ToGA) trial evaluated trastuzumab in combination with cisplatin and fluororyrimidine based chemotherapy in the first line setting [9]. Median overall survival (OS) was improved significantly for patients treated with trastuzumab with the greatest margin of benefit seen in those patients with high levels of HER2 overexpression (IHC 2+ or 3+, FISH positive) and as a result trastuzumab is a standard of care for patients with HER2 positive disease. Treatment with second-line chemotherapy is also now well established, with randomised studies of irinotecan, docetaxel and paclitaxel all demonstrating a survival advantage over best supportive care alone, in general yielding approximately a six week gain in medial overall survival [10,11]. Finally, the vascular endothelial growth factor receptor-2 (VEGFR-2) antibody ramicurumab has comparable efficacy to cytotoxic chemotherapy as a single agent in previously treated patients, and additive benefits when used in conjunction with paclitaxel [12,13]. However, despite these recent improvements in outcomes, less than 15% of patients with advanced gastroesophageal cancer live for more than two years, and there is a clear unmet need for more effective treatments.

The anatomical distribution of gastric cancer subtypes, reflective of differences in underlying aetiology, is also associated with distinct molecular subtypes [6]. The recent Cancer Genome Atlas research network (TCGA) study provides comprehensive molecular classification of gastric cancer [14]. Four subtypes are described; these are Epstein Barr virus (EBV) positive, microsatellite unstable (MSI), genomically stable (GS) and chromosomal instability (CIN) tumours. Notably, EBV-associated tumours are associated with elevated PD-L1/2 expression, which make this subtype attractive for immunotherapy treatment targeting PD-1 and its ligands. Additionally, microsatellite unstable tumours have a hypermutated phenotype which has been associated with to high response rates to immunotherapy in non-gastric cancer specific clinical trials [15]. However, although the interaction between non-EBV, non-MSI gastric cancer subtypes and immunotherapy is not known, this does not imply that the lack of known specific targets means that this approach could not be successful for these subtypes[16].

## 3. Immunotherapy–Basic Premises

**Figure 1:**
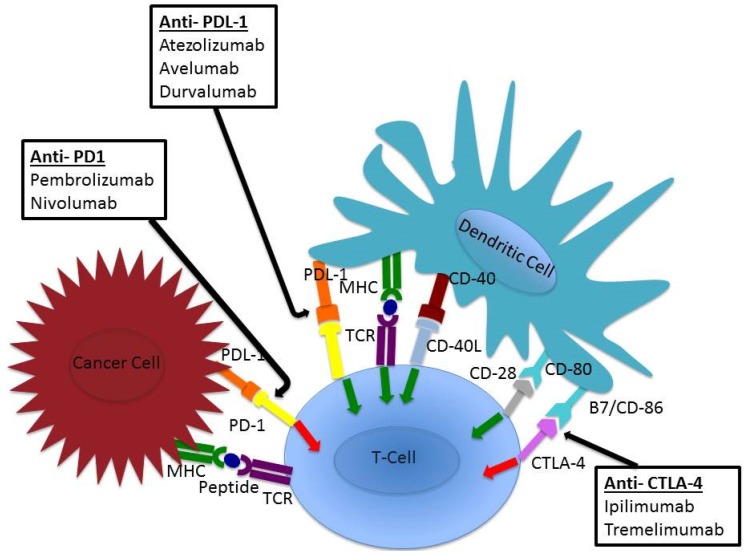
Immune checkpoints receptors and ligands on T cell, dendritic cell and tumour cell surfaces. T cell inhibitory signalling demonstrated with red arrows, stimulatory signalling demonstrated with green arrows. Site of action for developed Anti-PD-1, Anit-PD-L1 and Anti-CTLA-4 antibodies shown with black arrows.

In order to activate a specific anti-tumoural response, T-cells must be presented by their cognate peptide at the T-cell receptor by a major histocompatibility complex on an antigen presenting cell. (Figure 1) These interactions are governed by the interaction of inhibitory and co-stimulatory molecules between T cells and tumour cells, such as CTLA-4/Cluster of differentiation (CD)-28 and PD-1 and its ligands PDL-1/2 [17]. CTLA-4 is an inhibitory receptor and is activated by binding CD80 or CD86 on antigen presenting cells. It competes to bind CD80/86 with CD28, a T cell co-stimulatory protein. However, unlike CD28, which stimulates the T cell to respond, CTLA-4 inhibits T cell activation. Therefore, inhibition of this inhibitory circuit leads to T-cell activation [18]. PD-1 is another T-cell inhibitor molecule which is expressed on activated T-cells and which functions by binding to PD-L1 and PD-L2 on the antigen presenting cell, which inhibits T cell migration, proliferation and also effector functions including cytokine secretion. Importantly, expression of PD-L1 is also found on natural killer cells (NK) cells, dendritic cells, macrophages and mast cells and can be induced by inflammatory cytokines in tumour cells of various types. Therefore, PD-1 blockade may act beyond the early immune response in lymphoid tissues and affect the late response at other sites. These mechanisms have been exploited in cancer immunotherapy with promising results. We will discuss their role in gastroesophageal cancer treatment.

## 4. Interaction between Immune Status and OG Cancer/Outcomes

The presence of tumour infiltrating lymphocytes (TILs) has been associated with improved outcomes in melanoma, colorectal and breast cancers [19,20,21]. In gastric cancer, several studies suggest improved survival for patients with high levels of tumour infiltrating lymphocytes [22,23,24,25]. However, this may vary based on the exact lymphocyte subset assessed and the tumour compartment in which it is measured (tumour vs. stroma), and also the presence of immune stimulatory factors such as EBV and microsatellite instability. In the context of PD-L1 expression, the presence of high level CD8+ TIL infiltrate may be a negative prognostic marker, as demonstrated in by Thompson et al. who examined 34 resected gastroesophageal tumours. In this cohort 12% of samples demonstrated cell membrane PD-L1 expression, however expression of PD-L1 was more common in the immune stroma at 44% [26]. PD-L1 expression was found to correlate with CD8+ T cell density in tumours and immune stroma, suggesting an active adaptive immune response. In this study, patients with increased tumoural PD-L1 expression and CD8+ T cell density in tumour or stroma had worse progression-free survival and overall survival rates. More work is needed to fully evaluate the relationship between PD-L1 expression and T-cell activity as prognostic and predictive biomarkers in gastroesophageal cancer.

## 5. Checkpoint Inhibition

Targeting the immune checkpoint pathways of CTLA4, PD-1 and PD-L1 has been remarkably successful in melanoma, non-small cell lung cancer (NSCLC) and urothelial cancers, and is currently being investigated extensively in clinical trials for patients with gastroesophageal cancer [1,2,27,28].

Two anti-CTLA-4 antibodies, ipilimumab (Yervoy®, Bristol-Myers Squibb) and tremelimumab, (CP-675,206, Astra-Zeneca) have been assessed in gastroesophageal cancer, the former is licensed for use in melanoma. A phase 2 study of tremilimumab in the second line treatment of unselected advanced gastric and oesophageal cancer showed a response rate of 5% in 18 patients, with a median OS of 4.8 months [29] (Table 1). The sole responder was reported to continue on treatment at 32.7 months suggesting a durable response in excess of survival expected in this setting. The study also demonstrated patients who had proliferative carcinoembryonic antigen (CEA) responses had higher mOS (17.1 vs. 4.7m) than those who did not have a similar response. A second phase 2 study (NCT01585987) assessed the efficacy of ipilimumab as sequential or maintenance treatment immediately after first line chemotherapy in unresectable or metastatic gastric and gastroesophageal cancer compared to best supportive care [30]. Patients in the treatment group received 4 doses of ipilimumab followed by three monthly doses as maintenance until disease progression, after completion of their first line of chemotherapy. The preliminary results of this study were recently presented; 57 patients were treated in each arm of the study, and the majority of patients in the best supportive care arm were treated with chemotherapy. No difference was demonstrated in median overall survival between the two arms (12.1 months ipilimumab vs. 12.7 months best supportive care).

In contrast to the relatively disappointing results demonstrated with ipilimumab, the anti-PD-1 monoclonal antibody pembrolizumab (Keytruda®, Merck) was associated with encouraging response rates in gastric cancer [3]. In a global phase Ib study patients with gastric adenocarcinoma were screened for PD-L1 expression for study entry. Forty percent of patients were PD-L1 positive, and thirty-nine patients were treated with pembrolizumab, many of whom were treatment refractory. Eight patients demonstrated radiological responses (objective response rate 22%), and half of responding patients had not experienced disease progression at the time of study publication. In contrast to many second line chemotherapies, treatment with pembrolizumab was well tolerated. Toxicities were as follows: fatigue in seven (18%) patients, hypothyroidism in five (13%) patients, itch in five (13%) patients, and arthralgia in four (10%) patients, all of which have previously been described with pembrolizumab therapy. Grade 3 or 4 treatment-related adverse events occurred in five (13%) patients; one grade 3 pemphigoid; grade 3 peripheral sensory neuropathy; grade 3 fatigue, grade 3 hypothyroidism, and grade 4 pneumonitis. However, no patient discontinued therapy as a result of pembrolizumab related immune mediated toxicity. Of significant relevance for future development of PD-1 therapies in gastric cancer patients was that despite a requirement for PD-L1 positivity for study entry, on re-biopsy, eight of thirty five patients with a second biopsy were PD-L1 negative. Three factors may have affected this result. Firstly, a different PD-L1 assay was used for study entry and repeat testing. Secondly, PD-L1 status may change as a result of prior chemotherapy, and thirdly, PD-L1 status may be heterogeneous throughout the tumour, such intratumoural heterogeneity of biomarker expression is well documented in gastric cancer patients [31]. Whatever the reason, careful exploration of PD-L1 status and its relatedness to response to immunotherapies in patients with gastric cancer will be required moving forward.

The CHECKMATE 032 study assessed the efficacy of another anti-PD-1 monoclonal IgG4 antibody, nivolumab (Opdivo®, Bristol-Myers Squibb) in a PD-L1 biomarker unselected gastric population [32]. Nivolumab was approved in December 2014 by the Food and Drug Administration (FDA) for the treatment of metastatic melanoma, more recently for NSCLC, and has also shown promising activity in renal cell carcinoma. In Checkmate 032 59 patients were treated with nivolumab as a single agent, and treatment was associated with a response rate of 12%, and a median duration of response of 7.1 months in responders. Response rates in PD-L1 positive and negative patients were 18% and 12%, respectively. However, in view of the variability in PD-L1 expression demonstrated in the KEYNOTE 012 study, it might be reasonable to question whether the PD-L1 negative patients were truly biomarker negative. The initial results from the Checkmate032 study demonstrated a toxicity profile for single agent nivolumab comparable to that seen in other tumour types. Treatment-related events of any grade occurred in 66% of patients, 14% experienced grade 3 or 4 toxicity and no treatment-related death occurred. The most common grade 3 events seen were elevated aspartate aminotransferase (5%) and alanine aminotransferase (ALT) (3%). Other observed toxicities included pneumonitis, fatigue, diarrhoea, vomiting and hypothyroidism.

The combination of nivolumab in combination with ipilimumab has been associated with increased response rates and progression-free survival in patients with metastatic melanoma, in particular in PD-L1 negative patients [1]. The same combination in gastric cancer (at two separate dose levels) was assessed in separate arms of the Checkmate 032 study [32]. Nivolumab 1 mg/kg plus ipilimumab 3 mg/kg every three weeks for four cycles followed by nivolumab as a single agent was associated with a response rate of 24%, whereas nivolumab 3 mg/kg plus ipilimumab 1 mg/kg for four cycles on the same schedule followed by nivolumab as a single agent was associated with a response rate of 9.6%. Incremental benefits in terms of response for PD-L1 positive vs. PD-L1 negative patients were also seen for combination therapy. Grade 3 or greater AEs were seen in 27%–45% of patients treated with combination therapy which was higher than the rate associated with nivolumab therapy alone in the same patient group. Combination therapy is now under investigation in a randomised controlled trial. As studies proceed assessing the efficacy of combination immunotherapy regimens, it will be important to consider the safe management of grade 3 and 4 toxicities in this patient group. For example, in the phase 3 trial of combined ipilimumab and nivolumab in untreated melanoma the rate of immune mediated adverse events of any grade was 82.1% in the nivolumab group and 95.5% in the combination group whereas rates of grade 3 and 4 toxicities were 16.3% and 55% respectively. Therefore although there were no treatment-related deaths, close monitoring of toxicity and early intervention to manage this is clearly warranted.

## 6. Anti-PD-L1 (Atezolizumab, Avelumab, Durvalumab)

Several anti-PD-L1 therapies have been investigated in gastric cancer; Avelumab is a humanised anti-PD-L1 monoclonal antibody under investigation in a number of clinical trials. A phase 1b study (NCT01772004) was reported at ASCO 2016 by Chung et al in gastric and gastroesophageal junction adenocarcinoma patients who received avelumab after progression on prior therapy (>2nd line) (62 pts) and as switch maintenance (89 pts) following first line chemotherapy [33]. Response rates to avelumab have been modest in both settings; however, durability of responses has also been demonstrated (5/14 responses were >40 weeks). Again, the proportion of patients responding to avelumab was higher in those with PD-L1 positive cancers. Based on results from the phase I trial of durvalumab (MEDI4736), an anti-PD-L1 monoclonal antibody also suggested activity in gastric cancer, and several clinical trials are further investigating this compound in this disease [34]. Overall, response rates to anti-PDL1 therapy appear to be lower than to anti-PD-1 for patients with gastric cancer, and it is possible that enhancing response rates by using a combination approach such as mitogen-acivated protein kinase (MEK) inhibition plus anti-PDL1 therapy in colorectal cancer may be required in order to further develop this class of compound [35].

## 7. Anti-PD-1 and PD-L1 Therapy in Esophageal Adenocarcinoma

Many of the clinical trials assessing checkpoint inhibitors in gastroesophageal cancer have been conducted separately for patients with gastric and gastroesophageal cancer. This is understandable; however, many of the esophageal studies have also recruited squamous cell carcinoma, which may have a differential response profile to adenocarcinoma for reasons of underlying disease aetiology and which will not be further discussed here. The efficacy of pembrolizumab in oesophageal adenocarcinoma appears to be comparable to PD-L1 positive gastric cancer in the KEYNOTE 028 study. In this small cohort (*n* = 5), two patients with adenocarcinoma responded to therapy (Overall response rate (ORR) 40%) [36].

## 8. Biomarkers Associated with Response to Checkpoint Inhibitor Therapy in Gastroesophageal Cancer

The association of PD-L1 expression and response to immunotherapy is complex and not fully understood [37]. Furthermore, scoring criteria and antibody use are inconsistent across clinical trials. This is further complicated by the issue of heterogeneity of biomarker expression in gastric cancer [38]. As limited published data are currently available, assessment of the interaction of PD-L1 status and chemotherapy in ongoing large randomised trials will be essential in order to fully elucidate this relationship.

Gene expression profiling in melanoma has identified a signature associated with response to the anti-PD-1 inhibitor pembrolizumab [39]. Interestingly, when this signature was applied to gastric cancer patients treated in the KEYNOTE 012 study there appeared to be a trend towards improved survival in this separate patient group [3,40]. If validated in a larger patient group, this provides an important proof of concept for a tissue of origin-independent predictive marker for response to anti-PD-1 therapies. Another gene expression study provides a potential insight into regional variation in gastric cancer (GC) prognosis and response to biological therapies. Lin et al. investigated the gene expression profiles in Asian and non-Asia gastric adenocarcinomas; they found enrichment of T-cell expression signatures in non-Asian GCs [41]. Confirmatory immunohistochemistry analysis supported enrichment of most T-cell markers in non-Asian populations, with notable increased expression of the macrophage marker CD68. In multivariate analysis, only CD68 and CD3 expressions were independently associated with survival, and a high CD68/CD3 ratio was predictive of worse overall survival. Non-Asian patients were more likely to have high CD68/CD3 ratios consistent with non-Asian gastric cancers having poorer prognosis. In addition, CD68 macrophages and CD4 T cells are pro-angiogenic and therefore, variation in expression of these markers may account for geographical variability in trial results with anti-angiogenic monoclonal antibodies, potentially suggesting a role for infiltrating T cells as immune biomarkers for anti-angiogenic therapies.

Microsatellite instability (MSI) occurs as a result of defective mismatch repair [42]. Microsatellites are small, repetitive DNA sequences distributed in the human genome. When mutations occur in mismatch repair genes, such as human mutL homolog 1 (hMLH1) and human mutS homolog 2 (hMSH2) hMSH2 or they are silenced epigenetically, then replication errors within these microsatellite sequences cannot be repaired, producing a hypermutated phenotype. In gastric patients, MSI occurs in up to 22% of patients and is associated with female gender, older age and with distal, well-differentiated adenocarcinoma or intestinal type and with a lower stage at presentation [14]. Mismatch repair deficiency may have prognostic and predictive value; patients with mismatch repair deficient (MMRd) or MSI tumour have improved survival following surgical resection compared to patients with mismatch repair proficient or microsatellite stable tumours, and several studies suggest that the benefit of peri-operative chemotherapy might be less in patients with microsatellite unstable tumours [43,44,45,46,47,48,49,50].

Mismatch repair deficiency leading to a hypermutated phenotype and high levels of neo-antigen presentation is associated with an enhanced response to anti-PD-1 therapy across tumour types [51]. As up to 22% of gastric cancers display microsatellite instability, checkpoint blockade may be an attractive potential therapy for these patients [14]. In Keynote-012, four patients with mismatch repair deficient gastric cancers were treated with pembrolizumab, and two of these patients demonstrated a radiological response (ORR 50% for MMRd tumours) [3]. Thus, although mismatch repair deficiency is associated with increased response rates, it does not have a perfect positive predictive value.

Helicobacter pylori (*H. pylori*) is believed to cause up to fifty percent of gastric cancers and accounts much geographical variation in incidence [5,52]. Inflammation occurring secondary to *H. pylori* infection alters the gastric microenvironment and can accelerate neoplastic cell transformation and immune cell infiltration. The risk of gastric cancer in patients with *H. pylori* infection is driven by bacterial factors including secreted toxins (e.g. Vacuolating cytotoxin (Vac)) and functional proteins that alter cell structural integrity and induce inflammation (e.g. Cytotoxin-associated gene A (CagA)) [53]. Furthermore, *H. pylori* infection increases the levels of DNA methylation of genes, including tumour suppressors, and the induction of inflammation related genes, e.g. Tumour Necrosis Factor (TNF)-alpha [54]. Importantly, infection with *H. pylori* induces a T-cell response in gastric mucosa, but also increased expression of PD-L1, which is concurrently induced by *H. pylori* infection, leading to T-cell anergy. It might be therefore hypothesised that *H. pylori*-driven tumours might be more likely to respond to checkpoint inhibitor therapy; however, this is not yet proven. Furthermore, with respect to primary prevention of gastric cancer using an immunotherapy approach, development of vaccines against *H. pylori* could reasonably decrease the incidence of *H. pylori*-associated gastric cancer in much of the endemic areas of East Asia and other less developed countries [55].

Epstein Barr virus-mediated gastric cancers represent a unique molecular subgroup in the TCGA classification, accounting for 9% of gastric cancers [14]. This subset is characterised by PD-L1 and PD-L2 amplification, high levels of PD-L1 expression and immune infiltrate on tumour and immune cells, indicating another subset of gastric cancers which may respond well to checkpoint inhibitor therapy. EBV positive tumours such as Hodgkin lymphoma have demonstrated very encouraging responses to anti-PD-1 therapy; therefore, it is hypothesised that this might also occur in gastric cancer [56]. However, limited data are available on the interaction between EBV status and checkpoint inhibitor therapy in gastric cancer; this may be because EBV status (in common with MSI) is a powerful positive prognostic indicator in resected patients who may not go on to develop metastatic disease, thus limiting their exposure to trials of immunotherapies [57].

## 9. Vaccines, Adoptive T Cell Transfer, CAR T Cells

Vaccine therapies seek to exploit cellular immune responses to cancer antigens which may be “self” e.g. CEA, HER2 or “foreign” e.g. mutant RAS, mutant p53 or human papilloma virus (HPV), EBV. Such antigens may be delivered to the host immune system as peptides, proteins, or via dendritic cells. Dendritic cells which are antigen treated act as powerful activators of the immune response through presentation of antigen to T-cells. In order to enhance the immune response, antigens are commonly delivered in combination with adjuvants and/or cytokines such as interleukins or granulocyte macrophage colony-stimulating factor (GM-CSF). Tumour testis antigens such as melanoma associated antigen (MAGE)-3 and NY-ESO-1 are expressed in gastrointestinal tumours and testis, but not in normal tissue [58,59]. Use of NY-ESO-1 vaccines in oesophageal cancer patients led to CD4 and CD8 T-cell responses and tumour regression in one study, and other studies also confirm immune responses [60,61,62,63,64]. The gastrin peptide has also been targeted in a randomised phase II clinical trial in combination with cytotoxic chemotherapy [65]. Sixty-nine percent (65/94) of patients developed significant anti-gastrin antibodies and median survival was significantly longer in immune responders than non-responders (10.3 months vs. 3.8 months; *p* < or = 0.0001). Targeting the angiogenic pathway has also been associated with some activity in gastric cancer; in one trial (n = 22), a vaccine against human leucocyte antigen (HLA)-A24-restricted human vascular endothelial growth factor receptor 1 (VEGFR1)-1084 and VEGFR2-169 in combination with chemotherapy was associated with a median time to progression of 9.6 months and a median overall survival of 14.2 months [66]. Eighty-two percent of patients displayed immune responses against VEGFR1 and 2; however, only those with an immunological response to the VEGFR2-169 peptides showed statistically significantly improved survival.

Dendritic cells pulsed with tumour cell antigens have produced some initial promising result in gastric cancer. In one early study, Sadanaga et al. reported that MAGE-A3 peptide pulsed dendritic cells (DCs) were able to induce peptide specific T cell responses and minor tumour regression in some patients, and Kono et al. observed a tumour regression in one of nine patients treated with Her-2 (p369) pulsed dendritic cells [67,68]. One challenge of this approach is that dendritic cell therapy efficacy is short lived due to removal of dendritic cells by activated CD8+ lymphocytes and development of adjunctive therapies may be required in order to enhance this.

Amplification of patient derived T-cells ex vivo followed by re-infusion has been successful in melanoma; however, few studies have been conducted in patients with gastric cancer. In gastric cancer, Kono et al assessed the efficacy of adoptive immunotherapy with expanded patient-derived tumour associated lymphocyte lines in conjunction with chemotherapy [69]. Twenty-two patients were treated with adoptive autologous T cells plus chemotherapy and compared to twenty-two were treated with chemotherapy alone. Median survival was 8.5 months for the control group and 11.4 months for patients treated with adoptive T-cell therapy plus chemotherapy (*p* = 0.05). However, many patients will not develop T-cells which are specific for tumour antigens, limiting the applicability of this approach.

Chimeric antigen receptor (CAR) expressing T-cells have been associated with outstanding results in selected haematological malignancies [70,71]. These are T-cells which are genetically modified with addition of chimeric antigen receptor, which includes an antibody-based external receptor structure and cytosolic domains that encode signal transduction in the T cell [72]. The CAR external receptor directs T cells to specific tumour-associated antigens on malignant cells. CAR-T cells are quicker to produce in vitro and are therefore more practical than tumour infiltrating lymphocyte transfer. Furthermore, CAR-T cells are not restricted by HLA type and react to a wider range of molecules as they recognise any cell surface antigen, including proteins, carbohydrates and glycolipids. Antigens of interest for patients with gastric cancer include CEA and ERBB2, a Phase 1 study using CEA-targeted CAR T cells in CEA positive gastric, lung, breast, pancreatic and colorectal cancer (NCT02349724) is ongoing [73].

## 10. Impact of Next Generation Sequencing and Computational Biology on Immunotherapy

In future, selection of patients for immunotherapy may move beyond current strategies such as immunohistochemical tests such as PD-L1 or mismatch repair protein assessment. Neo-antigens may be derived from either driver or passenger mutations and are a driver of tumour infiltrating lymphocyte immune response [74]. Targeting tumour-specific neoantigens is attractive for a number of reasons: as they are expressed only by tumour cells, the risk of autoimmune toxicity with immunotherapy is reduced and additionally T-cells directed towards tumour specific antigens do not undergo thymic selection and therefore are of high affinity and increased cytotoxic ability [75,76]. Routine analysis of personal neo-antigens was not realistic until the recent evolution in high throughput next generation sequencing (NGS) technology. Using NGS, patient HLA subtype and mutational profile can be extracted and combined using epitope prediction algorithms to predict neoantigens [77]. However, although MHCI peptide binding can be predicted with reasonable accuracy, not all bound peptides are processed which is a requirement for antigen presentation, and algorithms predicting peptide processing lag behind those predicting epitope binding [78]. However, identification of neoantigens using NGS in mouse melanoma models with subsequent production of peptide vaccines based on these led to extended survival in mice with B16 tumours, providing proof of concept for a personalized immunotherapy approach in vivo [79]. In humans, a trial in three resected melanoma patients in the adjuvant setting demonstrated that vaccination with neoantigen derived peptides resulted in neo-antigen specific T cell generation; however, as patients had no active melanoma, T-cell specific tumoural responses were not seen; several trials are investigating this approach in patients with metastatic disease (NCT02035956, NCT 01970358).

A second approach to predicting the efficacy of immunotherapy treatments is monitoring of the T-cell receptor (TCR) repertoire before and after immunotherapy treatment [80]. The usual methodology for this is to perform TCR profiling, which amplifies DNA from the TCR β-chain CDR3 locus using PCR (polymerase chain reaction) primers, followed by next generation sequencing; however, novel approaches using computational biology have negated the requirement for pre-designed primers and allow de novo construction of the CDR3 sequences derived from TCR locus transcripts in paired-end RNA-seq data [81]. Application of this method to TCGA datasets across tumour types identified the frequent concurrent presence of specific tumor mutations and CDR3 sequence motifs which led to the identification through HLA epitope prediction of a putative immunogenic mutation in PRAMEF4. Thus, knowledge of the immunogenic mutanome can be enhanced via analysis of tumour or via the T-cell repertoire.

## 11. Conclusions and Future Directions

Improvements in systemic therapy for gastroesophageal cancer are urgently required, and early data suggest that immunotherapy may be helpful for a proportion of gastroesophageal cancer patients. At this time, checkpoint blockade with PD-1 or anti-PD-L1 either alone or in combination with anti-CTLA4 therapy has demonstrated the most promise for patients with gastroesophageal cancer. Integration of these therapies with currently used treatments such as chemotherapy and monoclonal anti-bodies such as trastuzumab and ramucirumab is yet to be optimised. Trials of anti-PD-1 therapy in combination with chemotherapy, and randomised against chemotherapy are ongoing. Given the relatively short survival of gastroesophageal cancer patients, in particular in the second line setting, the relatively low response rates seen with immunotherapy and prolonged time to response this treatment may not be optimal for unselected patient populations. Furthermore, first line chemotherapy for gastric cancer, in particular with triplet combination therapy, is relatively myeloablative, and the interaction between this and checkpoint inhibitor therapy remains to be elucidated. Additionally, specific chemotherapy drugs, in particular weekly paclitaxel and oxaliplatin may lead to immunogenic cell death with exposure of tumour antigens and these may have synergy with checkpoint inhibitors. Such chemotherapy may increase tumour antigen and costimulatory molecule expression, and downregulate coinhibitory signalling [75]. Emerging data also suggest that in colon cancer, use of MEK inhibitors in conjunction with checkpoint blockade may increase the efficacy of immune directly therapies in microsatellite stable (MSS) *RAS* mutant colorectal cancer; this strategy could also conceivably apply to gastric cancers, many of which display RAS-MEK-ERK pathway activation through receptor tyrosine kinase amplification [35]. Finally, use of immunotherapy with antibody therapy targeting HER2 or VEGFR2 may augment the effects of antibody directed cellular cytotoxicity, making this an attractive therapeutic option in conjunction with these established targeted therapies.

It is thus evident that more work is needed to determine how best to select patients for treatment, how to sequence this, and whether it should be combined with other agents. Exploration and validation of biomarkers associated with response to anti-PD-1 therapy in large scale trials is mandated in order to deliver the best value from novel therapies. Examination of the effect of microsatellite instability and EBV on the immunotherapy efficacy may yield insights which can be utilised in developing treatments for non-immunogenic tumours. Although data are currently limited, reporting of ongoing trials will lay the framework for new paradigms for treatment of this disease

## Figures and Tables

**Table 1:** Clinical trials investigating checkpoint inhibitors in gastroesophageal cancer.

Trial Phase	Drugs Tested	*N*	Median PFS (m)	ORR	Median OS (m)	1 Year OS
Phase II (28)	Tremilimumab	18	2.8	6%	4.8	33%
Phase II (29)	Ipilimumab	57	2.7	NR	12.7	NR
Phase Ib (3)	Pembrolizumab *	39	1.9	22%	11.4	NR
Phase I/II(31)	Nivolumab	59	NR	14%	5.0	36
	Nivolumab 1 mg/kg, Ipilimumab 3 mg/kg	49	NR	26%	6.9	34
	Nivolumab 3 mg/kg, Ipilimumab 1 mg/kg	52	NR	10%	4.8	NR
Phase Ib (32)	Avelumab 1st line maintenance	89	12 weeks	9%	NR	NR
	Avelumab 2nd line	62	6 weeks	9.7%	NR	NR

m (months); NR – not reported; * PD-L1 selected.
